# Cardiac events during treatment with proteasome inhibitor therapy for multiple myeloma

**DOI:** 10.1186/s40959-017-0023-9

**Published:** 2017-06-01

**Authors:** John H. Chen, Daniel J. Lenihan, Sharon E. Phillips, Shelton L. Harrell, Robert F. Cornell

**Affiliations:** 1 Department of Medicine, Division of Hematology-Oncology, Vanderbilt University Medical Center, 2220 Pierce Avenue, 777 PRB, Nashville, TN 37232 USA; 2 Department of Medicine, Division of Cardio-Oncology, Vanderbilt Heart & Vascular Institute, 1215 21st Avenue South, 5209 MCE, Nashville, TN 37232 USA; 3 Department of Biostatistics, Division of Cancer Biostatistics, Vanderbilt University Medical Center, 2220 Pierce Avenue, 571 PRB, Nashville, TN 37232 USA

**Keywords:** Myeloma, Bortezomib, Carfilzomib, Proteasome inhibitors, Risk factors, Cardiotoxicity

## Abstract

**Background:**

Proteasome inhibitors (PI) bortezomib and carfilzomib are cornerstone therapies for multiple myeloma. Higher incidence of cardiac adverse events (CAEs) has been reported in patients receiving carfilzomib. However, risk factors for cardiac toxicity remain unclear. Our objective was to evaluate the incidence of CAEs associated with PI and recognize risk factors for developing events.

**Methods:**

This was a descriptive analysis of 96 patients with multiple myeloma who received bortezomib (*n* = 44) or carfilzomib (*n* = 52). We compared the cumulative incidence of CAEs using a log rank test. Patient-related characteristics were assessed and multivariate analysis was used to identify risk factors for developing CAEs.

**Results:**

PI-related CAEs occurred in 21 (22%) patients. Bortezomib-associated CAEs occurred in 7 (16%) patients while carfilzomib-associated cardiac events occurred in 14 (27%) patients. The cumulative incidence of CAEs was not significantly different between agents. Events occurred after a median of 67.5 days on PI therapy. Heart failure was the most prevalent event type. More patients receiving carfilzomib were monitored by a cardiologist. By multivariate analysis, a history of prior cardiac events and longer duration of PI therapy were identified as independent risk factors for developing CAEs.

**Conclusions:**

AEs were common in patients receiving PIs. Choice of PI did not impact the cumulative incidence of CAEs. Early involvement by a cardiologist in patients at high risk for CAEs may help to mitigate the frequency and severity of CAEs.

## Background

Multiple myeloma (MM) is a clonal disorder of terminally differentiated plasma cells that accounts for approximately 13% of hematologic malignancies [1]. Clinical features of MM include hypercalcemia, anemia, renal insufficiency, lytic bone disease and increased risk of infection. Proteasome inhibitors (PIs) have become cornerstone therapies for management of MM [2–6]. These agents bind to the constitutive 26S proteasome, causing accumulation of protein byproducts within plasma cells and subsequent apoptosis [7, 8]. Bortezomib is used predominately in the front line setting, while second-generation carfilzomib is used in the relapsed/refractory setting.

Cardiac toxicities associated with carfilzomib identified in earlier studies include hypertension, arrhythmia, heart failure, ischemic heart disease and cardiomyopathy [9, 10]. These carfilzomib-associated toxicities were confirmed to occur at a higher incidence compared to bortezomib in the ENDEAVOR trial [11]. While a strong signal for cardiac toxicity has not been associated with bortezomib use, several case reports [12–16] have described CAEs in patients receiving this agent.

Patients with MM have a 54–74% risk of cardiovascular disease at baseline [1, 9, 10, 17]. Reasons for this increased burden include advanced age at diagnosis (median 62 years), exposure to various chemotherapies including autologous stem cell transplantation, and disease specific complications such as cardiac amyloidosis and renal insufficiency. The primary objective of this research was to identify cardiac event risk factors for patients receiving PI-based therapies for multiple myeloma.

## Methods

This was a descriptive analysis of 96 consecutive MM patients receiving PI-based therapies at Vanderbilt University Medical Center from 2011 to 2014. MM disease risk was determined by conventional metaphase cytogenetic data or fluorescence in situ hybridization data according to the Mayo Stratification for Myeloma and Risk-Adapted Therapy [18]. A line of therapy was defined as planned initial therapy in patients. For example, planned induction chemotherapy followed by autologous stem cell transplantation followed by maintenance therapy was considered one line of therapy. Estimated 10-year atherosclerotic cardiovascular disease (ASCVD) risk was calculated using the Pooled Cohort Equations; this represented their risk of myocardial infarction, coronary artery disease death, fatal or nonfatal stroke. Patients with light chain (AL) amyloidosis (biopsy proven or clinically suspected) were excluded.

Cardiac adverse events (CAEs) were evaluated only while patients were on active therapy with either carfilzomib- or bortezomib-based treatment. CAEs were graded according to the Common Terminology Criteria for Adverse Events version 4.0. Heart failure was characterized by at least 1 item in 2 out of the 3 following categories: symptoms (paroxysmal nocturnal dyspnea, shortness of breath, swelling, orthopnea, weight gain), physical findings (jugular venous distention, crackles, peripheral edema, S3 gallop), and diagnostic tests (pulmonary edema on chest radiograph, elevated B-type natriuretic peptide). Severity of heart failure events was defined as symptoms with mild to moderate activity (grade 2), symptoms at rest or with minimal exertion requiring intervention (grade 3), or life-threatening consequences requiring urgent intervention (grade 4). Acute coronary syndrome was defined as at least 2 out of 3 clinical findings of chest pain, troponin I elevation, and ischemic electrocardiographic changes. Sudden cardiac death was defined as death within 24 h of PI therapy without obvious cancer progression. Symptomatic arrhythmias included atrial fibrillation or flutter requiring treatment. Arterial and/or venous thromboembolism were determined by imaging. Pulmonary hypertension was defined as tricuspid regurgitation >3 m/s. Grade III hypertension was defined as systolic BP ≥160 mm Hg or diastolic BP ≥100 mm Hg requiring more than one drug or more intensive therapy than previously used. Grade IV hypertension was defined by life-threatening consequences (e.g. malignant hypertension, transient or permanent neurologic deficit, hypertensive crisis) requiring urgent intervention.

### Statistical analysis

The primary objective was to determine risk factors for development of CAEs in patients receiving PI-based therapy. Patient-related factors were compared between patients with and without CAEs using the Wilcoxon rank-sum test for continuous variables and chi-square test for categorical variables. Cumulative incidence of CAEs was compared between bortezomib and carfilzomib using the log-rank test. Logistic regression multivariate analysis was performed to identify risk factors for developing PI-related CAEs. The variables considered in this analysis were a prior history of cardiac events, use of antithrombotic (antiplatelet or anticoagulant) medications, and cumulative number of days on therapy. For days on therapy analysis, various time intervals were tested. Six-month time intervals were selected based on confidence interval and reproducibility. A statistical significance level of .05 was used throughout. Computations were performed using the statistical package R (version 2.3.1).

## Results

A total of 96 patients received PIs with 46% (*n* = 44) receiving bortezomib-based therapy and 54% (*n* = 52) receiving carfilzomib-based therapy. All patients in the carfilzomib cohort had prior exposure to bortezomib during previous lines of therapy; however, only bortezomib-related events in the bortezomib cohort and carfilzomib-related events in the carfilzomib cohort were included in the analysis. The cut-off date for collection of follow-up data was July 2015.

**Fig. 1:**
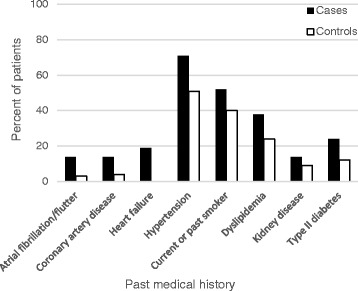
Baseline cardiovascular characteristics in patients receiving proteasome inhibitor therapy. Prevalence of cardiovascular risk factors in patients who developed cardiac adverse events (cases) versus those who did not (controls)

PI-related CAEs occurred in 22% (*n* = 21) of patients. Table 1 and Fig. 1 depict baseline characteristics grouped by patients who experienced CAEs (cases, *n* = 21) versus those who did not (controls, *n* = 75). More patients in the cases group had a prior history of atrial fibrillation/flutter (14% vs 3%, *P* = .03) or heart failure (19% vs 0%, *P* < .001) than in the control group. Additionally, patients in the cases group were more likely to be taking antithrombotic (52% vs 29%, *P* = .05), beta blocker (52% vs 15%, *P* < .001), lipid-lowering (67% vs 23%, *P* < .001), or loop diuretic (19% vs 5%, *P* = .04) medications. Lastly, number of days on PI therapy was greater in patients who experienced CAEs versus those who did not (407 vs 250, *P* = .02).

**Table 1:** Baseline characteristics in case control study of cardiac events associated with PI therapy

	Cases *N* = 21	Controls *N* = 75
Years of age, median (range)	63 (46–86)	59 (36–91)
Male, *n* (%)	11 (52)	51 (68)
Caucasian, *n* (%)	13 (62)	60 (80)
Current or past smoker, *n* (%)	11 (52)	30 (40)
Dyslipidemia, *n* (%)	8 (38)	18 (24)
Type II diabetes, *n* (%)	5 (24)	9 (12)
Kidney disease, *n* (%)	3 (14)	7 (9)
10-Year ASCVD risk >20%, *n* (%)	7 (37)	8 (14)
Prior cardiac event, *n* (%)	16 (76)	41 (55)
Atrial fibrillation/flutter, *n* (%)	3 (14)	2 (3)
CAD, *n* (%)	3 (14)	3 (4)
Heart failure, *n* (%)	4 (19)	0 (0)
Hypertension, *n* (%)	15 (71)	38 (51)
Venous/arterial thromboembolism, *n* (%)	3 (14)	4 (5)
Valvular disease, *n* (%)	2 (10)	1 (1)
ACE inhibitor or ARB use, *n* (%)	8 (38)	24 (32)
Antithrombotic use, *n* (%)	11 (52)	22 (29)
Beta blocker use, *n* (%)	11 (52)	11 (15)
Lipid lowering agent use, *n* (%)	14 (67)	17 (23)
Loop diuretic use, *n* (%)	4 (19)	4 (5)
Followed by cardio-oncologist	7 (33)	16 (21)
Durie Salmon stage III, *n* (%)	11 (52)	47 (63)
International Staging System III, *n* (%)	5 (24)	23 (31)
Cytogenetic/FISH high risk, *n* (%)	2 (10)	8 (11)
Received bortezomib only, *n* (%)	7 (33)	37 (49)
Received carfilzomib^a^, *n* (%)	14 (67)	38 (51)
Total days of PI therapy, median (range)	407 (38–1032)	250 (2–885)

*Abbreviations*: *PI *proteasome inhibitor, *ASCVD* atherosclerotic cardiovascular disease, *CAD* coronary artery disease, *ACE* angiotensin-converting enzyme, *ARB* angiotensin receptor blocker, *FISH* fluorescent in situ hybridization

^a^All patients previously received bortezomib

By multivariate analysis (Table 2), every 6-month interval of PI therapy was associated with higher incidence of CAEs (HR 1.94, 95% CI 1.21–3.09, *P* = .006). Additionally, a history of cardiac events prior to initiation of PI therapy predisposed to treatment-emergent CAEs (HR 3.48, 95% CI 1.03–12.8, *P* = .045). Antithrombotic use trended towards greater likelihood of CAEs but did not reach statistical significance.

**Table 2:** Multivariate analysis of clinical parameters influencing risk of cardiac adverse events

	OR	95% CI	*P*-value
Prior history of cardiac events			
No	1	−	.045
Yes	3.48	1.03–12.8	
Use of antithrombotic agents			
No	1	−	.056
Yes	3.66	0.97–8.79	
Duration of PI therapy	1.94	1.21–3.09	.006

*Abbreviations*: *OR* odds ratio, *CI* confidence interval, *PI* proteasome inhibitor

**Fig. 2:**
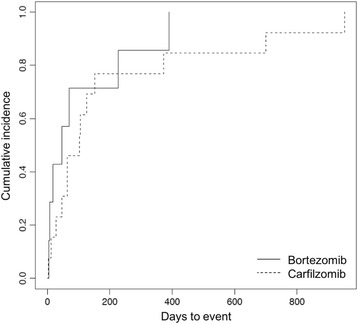
Cumulative incidence of cardiac adverse events. Cumulative incidence of cardiac adverse events associated with bortezomib (*solid line*) and carfilzomib (*dashed line*) in relation to duration of therapy shown in days

Cumulative incidence of CAEs was not significantly different between the 2 PI agents (log-rank test *P* = .41, Fig. 2). Bortezomib-related CAEs occurred in 7 (16%) out of 44 patients while carfilzomib-related CAEs occurred in 14 (27%) out of 52 patients. Carfilzomib users had significantly more prior lines of therapy (median, 2 vs. 0; P < .001) and were significantly more likely to be followed by a cardio-oncologist (35% vs. 11%, *P* = .008).

Events occurred after a median of 67.5 (range, 4–456) days on PI therapy. Grade 3 or 4 events accounted for 56% of CAEs. There were no cardiac arrests or deaths associated with PI therapy. In the setting of a cardiac event, planned PI therapy was permanently discontinued 29% of the time (43% of the time when a cardio-oncologist was involved, 21% of the time in the absence of cardiology care, *P* = .30). Heart failure was the most common type of CAE for both bortezomib and carfilzomib (Table 3). Four out of 13 (31%) heart failure events led to withdrawal of PI therapy. Nine out of 13 patients who developed symptomatic heart failure had preserved left ventricular ejection fraction (LVEF); 2 bortezomib users and 2 carfilzomib users experienced decline in LVEF to < 55% from previously normal LVEF.”

**Table 3:** Proteasome inhibitor related cardiac adverse events

Cardiac adverse event, *n*	Bortezomib (*n* = 44)	Carfilzomib (*n* = 52)	Total (*n* = 96)
Any type^a^	7	14	21
Heart failure	6	7	13
Grade 3 or 4	3	4	7
Systemic hypertension	1	3	4
Grade 3 or 4	1	3	4
Thromboembolism	1	2	3
Grade 3 or 4	0	0	0
Acute coronary syndrome	1	1	2
Grade 3 or 4	1	0	1
Atrial fibrillation/flutter	0	2	2
Grade 3 or 4	0	1	1
Pulmonary hypertension	0	1	1
Grade 3 or 4	0	1	1
Orthostatic hypotension	0	0	0
Sudden cardiac death	0	0	0

^a^Some patients had multiple events

## Discussion

While the mechanism of cardiotoxicity from these agents is not entirely understood, off-target proteasome inhibition is believed to cause accumulation of misfolded proteins within cardiomyocytes [19]. A number of in vitro and in vivo animal studies [20–29] have linked dysfunction of the ubiquitin-proteasome system to myocardial injury, cardiomyopathies, and atherosclerosis.

A high prevalence of cardiovascular disease is well-documented in the MM patient population, with 54–74% having a history of cardiac events at baseline [9, 10, 17]. However, risk factors for developing PI-related cardiotoxicity are not well established. Clarifying the risk factors for PI toxicity will allow providers to guide patient selection and facilitate risk reduction strategies.

We demonstrate that history of prior cardiac event was the most significant risk factor for developing PI-related CAEs. Specifically, a history of heart failure or atrial fibrillation/flutter was significantly more prevalent in patients who went on to develop CAEs versus those who did not. Patients who developed CAEs also had higher usage rates of antithrombotic (antiplatelet or anticoagulant), beta blocker, lipid lowering, and loop diuretic medications, which suggests a higher burden of baseline comorbidities in this group. Based on these findings, we recommend careful consideration of patients’ past medical history and preexisting cardiovascular risk factors with use of PI therapy.

Based on existing literature, we expected more CAEs in the carfilzomib cohort compared to the bortezomib cohort, especially since patients receiving carfilzomib were more heavily pretreated. Pooled safety data from 4 phase II clinical trials for carfilzomib demonstrated a 22% incidence of CAEs [9]. In a retrospective study of 130 carfilzomib users with relapsed/refractory multiple myeloma (RRMM), 26 (20%) were hospitalized due to CAEs [10]. Meanwhile, bortezomib-associated CAEs have been considered relatively uncommon. A meta-analysis of data from key phase II and III studies demonstrated a 2% incidence of bortezomib-associated high grade heart failure in both newly diagnosed and RRMM cohorts [30]. Additionally, the 2 PI agents were directly compared in the ENDEAVOR trial [11], which again demonstrated a higher cardiac event rate with carfilzomib compared with bortezomib. It is interesting that a cardiac substudy within this trial found higher rates of hypertension, heart failure, and pulmonary hypertension in the carfilzomib group.

In our data from a “real world” clinical setting, there was not a statistically significant difference in CAEs between patients receiving carfilzomib or bortezomib. Crucially, patients treated with carfilzomib were significantly more likely to receive co-managed care from a cardio-oncologist. We propose that routine monitoring by a cardiologist may have reduced the incidence and severity of CAEs in carfilzomib users. Furthermore, much of the observed cardiotoxicity is perhaps difficult to distinguish from baseline cardiovascular dysfunction in this at-risk population. We therefore recommend co-managed care as a general strategy in all MM patients for risk reduction by way of optimizing medical therapy and controlling comorbidities.

Other important factors to consider include timing of events and types of cardiac toxicity. In phase II clinical trials for carfilzomib [9], 12% of patients experienced CAEs within 1 day of initial dosing, and the incidence of events did not increase in later cycles. While there were CAEs in this series occurring within days of initial dosing, multivariate analysis exposed a significant positive correlation between duration of therapy and risk of events. However, we cannot be certain that this correlation reflects a treatment effect rather than a longer observation period. In the phase II carfilzomib [9] and phase III ENDEAVOR trials [11], hypertension occurred most frequently but heart failure was most likely to cause treatment discontinuation. Similarly, we found that heart failure and hypertension comprised the majority of CAEs in both bortezomib and carfilzomib users.

### Limitations

The study may have been underpowered to detect a significant difference in incidence of CAEs between carfilzomib and bortezomib. Due to the retrospective design of this study, assignment to treatment drug was nonrandomized. Since carfilzomib is often reserved for relapsed/refractory MM, all patients in the carfilzomib cohort had prior exposure to bortezomib and in general were more heavily pretreated. Therefore, cumulative effects or delayed toxicity from previous agents may account for a higher rate of CAEs in the carfilzomib cohort. Finally, we could not rule out alternative etiologies of cardiac dysfunction when attributing suspected CAEs to PI therapy, particularly when concomitant agents were used.

## Conclusion

Incidence of PI-related CAEs did not differ significantly between bortezomib and carfilzomib users; both agents were associated with substantial rates of cardiotoxicity. Prior history of cardiac events was associated with increased risk of developing PI-related CAEs. Longer duration of PI therapy correlated with greater likelihood of cardiotoxicity. Heart failure was both an important risk factor and subsequently the most prevalent type of PI-related cardiotoxicity. Close monitoring by a cardio-oncologist may reduce risk of CAEs.

## Abbreviations

ASCVD: Atherosclerotic cardiovascular disease
CAE: Cardiac adverse event
CI: Confidence interval
HR: Hazard ratio
LVEF: Left ventricular ejection fraction
MM: Multiple myeloma
PI: Proteasome inhibitor
RRMM: Relapsed/refractory multiple myeloma
